# Cytomegaloviruses: From Molecular Pathogenesis to Intervention

**DOI:** 10.3201/eid1911.131226

**Published:** 2013-11

**Authors:** Julian Tang

**Affiliations:** Alberta Provincial Laboratory for Public Health, Edmonton, Alberta, Canada.

**Keywords:** cytomegalovirus, viruses, basic science, clinical aspects, overviews, neonatal, pregnancy, transplant, immune response, pathogenesis, latency

Many health professionals rely on journal articles to keep up with advances in their field because textbooks are often 1–2 years out of date by the time they come to press, and are far more expensive than the occasional PDF downloaded from a university library Web site. This 2-volume text on cytomegalovirus (CMV) is costly and cites data from before 2012, but provides a solid foundation on which to apply new findings. Volume I is mainly focused on the basic science of and related animal experiments on CMV; volume II is aimed at the clinical reader, again with chapters on relevant animal model studies. Each chapter reads as a short review article, and is easily digestible. Many well-recognized experts in this field contributed content, which should be reassuring to the reader. The text and referencing style are easy to read and the figures and tables are illustrative and helpful. The volumes come in a compact size, making them convenient to carry, and also are downloadable as eBooks.

Volume I gives in-depth overviews of primate and murine CMVs, CMV metabolomics, miRNAs, and proteomics. Most of the chapters are dedicated to viral gene expression and function and virus interaction with human host cells, describing immune response, aspects of viral tropism, entry, pathogenesis, and latency. The terminology used in Volume I is specialized and may be difficult for readers who do not work in these fields.

Volume II covers essential clinical background: the epidemiology of CMV infections in pregnancy, CMV infections in solid and bone marrow transplants, CMV therapy and drug resistance, diagnostic methods, and vaccine development. Additional chapters describe the host immune response to CMV infection, and mechanisms of infection in specific targets, such as the placenta.

Chapters in both volumes are detailed and clearly written; the editors are to be commended on maintaining this standard. However, additional detail and discussion (i.e., pros and cons) about alternative targets for CMV PCR monitoring of transplant patients, such as pp65 antigen and pp67 mRNA versus DNA, would have been helpful. Also, an in-depth chapter on the characteristics of reinfecting or superinfecting strains of CMV in various clinical situations would be useful; such strains are frequently referred to without description throughout the clinical text. Perhaps one surprising omission on the clinical side is a chapter comparing and contrasting the various clinical guidelines for the treatment of CMV infections in transplant patients; this would highlight differences in how published data are interpreted.

Chapter II.23, Putative Disease Associations with Cytomegalovirus: a Critical Survey, explores the possible role of CMV as an etiologic agent for specific clinical syndromes, including glioblastomamultiforme, cardiovascular disease, and the role that CMV may play in immunosenescence. This makes fascinating and educational reading, especially in how the authors tease out the relevant (and irrelevant) evidence for and against these potential etiologic roles.

The price tag is considerable, although the 2 volumes can be purchased separately (http://www.horizonpress.com/hsp/supplementary/cmv2/cmv2-vol1-vol2.html). As a medical–clinical virologist, I find Volume II to be a useful reference text. Those working on the basic virology of CMV may consider Volume I a useful addition to their libraries. These volumes give comprehensive, yet succinct overviews of the current state of knowledge of many aspects of CMV and are detailed enough to satisfy most readers.

